# Retraction: CSF p-tau as a potential cognition impairment biomarker in ALS

**DOI:** 10.3389/fneur.2024.1392563

**Published:** 2024-03-04

**Authors:** Frontiers Editorial Office

The journal retracts the 01 November 2022 article cited above.

Following publication, concerns were raised regarding the validity of the data in the article. The authors failed to provide the raw data during the investigation, which was conducted in accordance with Frontiers' policies. Given the lack of raw data, the editors no longer have confidence in the findings presented. The article is therefore retracted.

This retraction was approved by the Chief Editors of Frontiers in Neurology and the Chief Executive Editor of Frontiers. The authors agree to this retraction.

Frontiers would like to thank the concerned reader who contacted us regarding the published article.

